# Structural ambiguity of the Chinese version of the hospital anxiety and depression scale in patients with coronary heart disease

**DOI:** 10.1186/1477-7525-4-6

**Published:** 2006-01-26

**Authors:** Wenru Wang, Violeta Lopez, David Thompson, Colin R Martin

**Affiliations:** 1 School of Medicine, Xi'an Jiaotong University, Xi'an, Shannxi, China; 2 Faculty of Medicine, Chinese University of Hong Kong, Hong Kong, China; 3 Nethersole School of Nursing, The Chinese University of Hong Kong, Hong Kong, China; 4 Department of Mental Health and Learning Disability, University of Sheffield, S63 7ER, UK

## Abstract

**Background:**

The Hospital Anxiety and Depression Scale (HADS) is a widely used screening tool designed as a case detector for clinically relevant anxiety and depression. Recent studies of the HADS in coronary heart disease (CHD) patients in European countries suggest it comprises three, rather than two, underlying sub-scale dimensions. The factor structure of the Chinese version of the HADS was evaluated in patients with CHD in mainland China.

**Methods:**

Confirmatory factor analysis (CFA) was conducted on self-report HADS forms from 154 Chinese CHD patients.

**Results:**

Little difference was observed in model fit between best performing three-factor and two-factor models.

**Conclusion:**

The current observations are inconsistent with recent studies highlighting a dominant underlying tri-dimensional structure to the HADS in CHD patients. The Chinese version of the HADS may perform differently to European language versions of the instrument in patients with CHD.

## Background

In China, economic transition, urbanization, industrialization and an aging population have quickly increased the incidence and prevalence of coronary heart disease (CHD) in the past decades [1]. CHD has been ranked among the top three causes of death in China [2]. Anxiety and depression are common psychological problems associated with a diagnosis of CHD [3-5]. Importantly, depression and anxiety have been linked with the morbidity and mortality of CHD [6]. Therefore, valid and reliable screening for clinically significant anxiety and/or depression is paramount in this clinical group.

The Hospital Anxiety and Depression Scale (HADS) [7] is a widely used, self-administered questionnaire specifically developed to detect anxiety and depression states in hospital and medical out-patient clinic settings. It is composed of two 7-item scales, one for anxiety and one for depression.

The original English version has been translated into and validated in many languages, including Chinese [8-12]. The Chinese version of HADS is a popular instrument for assessing psychological distress in clinical studies in China [5,12-14]. A number of studies have validated the Chinese version of this questionnaire both in Hong Kong (HK) and China [8,12,15]. Leung and colleagues [8] evaluated the psychometric properties of the Chinese version of HADS in 100 medical students. The results indicated factorial inconsistency with the English language version with a three-factor solution emerging. Despite this anomaly, the authors concluded the instrument was a valid Chinese translation. A more recent study of the Chinese version of the HADS [12] across a broad clinical range of in-patients again found three underlying factors to the instrument. These were interpreted as depression and two distinct factors of psychic anxiety and psychomotor agitation [12]. However, the utility of the instrument is based on the theoretical assumption of an underlying bi-dimensional (anxiety and depression) factor structure. Moreover, a underlying tri-dimensional structure of the HADS may have significant implications for both scoring and case detection accuracy [16,17].

A recent review [16] of the HADS has suggested that the instrument may in reality have an underlying tri-dimensional factor structure in CHD patients and other clinical groups. Recent investigations of the psychometric properties of the HADS in CHD patients support the notion that the instrument essentially comprises three, rather than two sub-scales [18-20]. One study [20] of Cantonese-speaking Chinese CHD patients in Hong Kong has also furnished compelling evidence for the tri-dimensionality of the HADS. Hong Kong Chinese invariably speak Cantonese whereas in the Xi'an province of China Mandarin is spoken. Due to the pictorial nature of Chinese writing, both Cantonese and Mandarin-speaking Chinese would be able to read the Chinese version of the HADS.

In Europe, the HADS has been applied extensively in the studies of patients with CHD as an index of both outcome and the effect of therapeutic intervention [21-23]. There have also been reports that the Chinese version of HADS may have some utility as a screening and assessment tool in patients with CHD [5]. However, the factorial structure of the Chinese version of the HADS has not been established in this clinical group with consequent implications for screening and case detection utility and accuracy [16]. The present study was designed to examine the underlying factor structure of the Chinese version of the HADS in a mainland Mandarin-speaking population of patients admitted to hospital with CHD.

## Methods

### Design

The study used a cross-sectional design. To address the research question confirmatory factor analysis methods were used on a pooled HADS data set from mainland Mandarin-speaking patients admitted to hospital with CHD.

### Statistical analysis

The factor structure of the HADS was determined using confirmatory factor analysis using Mplus version 3 [24]. The weighted least-square with mean and variance correction estimator (WLSMV) was used to evaluate model fit as this estimation method can be both used reliably with ordered categorical level data, and be used dependably with modest samples sizes. Seven models developed from HADS validity and psychometric studies were tested [7,17,25-28]. The characteristics of each model tested and item-factor loading characteristics are shown in Table 1. Confirmatory factor analysis represents a powerful statistical technique used to determine whether the number of factors and pattern of item-factor loadings is consistent with what would be expected by a priori theory. This represents a significant methodological advance over the more commonly used exploratory factor analysis where no prior assumptions of structure are explicitly made. Confirmatory factor analysis is a special case of structural equation modelling and is statistically and methodologically distinct from exploratory factor analysis. Though strictly speaking exploratory factor analysis should be used to determine the original factor structure of an instrument, and confirmatory factor analysis used to determine how well an a priori-defined factor structure fits data, it is common in the literature to see exploratory factor analysis used to investigate the factor structure of an instrument that has previously been investigated using exploratory factor analysis. Consequently, exploratory factor analysis is often found to be used where confirmatory factor analysis would be more desirable and more appropriate, this situation being widely observed in many previous studies of the factorial structure of the HADS.

**Table 1:** Characteristics of each factor model tested

**Model**	**Number of factors**	**Clinical population**	***n***	**Factor extraction method**^**#**^
Zigmond and Snaith (1983)	2	Medical	100	No factor analysis
Moorey et al. (1991)	2	Cancer	568	PCA
Dunbar et al. (2000)	3	Non-clinical	2,547^+^	CFA
Friedman et al. (2001)*	3	Depressed	2,669	PCA
Razavi et al. (1990)	1	Cancer	210	PCA
Caci et al. (2003)**	3	Non-clinical	195	CFA

	FLI1**	**FLI2**	**FLI3**	

Zigmond and Snaith (1983)	1,3,5,7,9,11,13	2,4,6,8,10,12,14	---------	
Moorey et al. (1991)	1,3,5,9,11,13	2,4,6,7,8,10,12,14	---------	
Dunbar et al. (2000)	1,5,7,11	2,4,6,7,8,10,12,14		
Friedman et al. (2001)*	1,7,11	2,4,6,8,10,12,14	3,5,9,13	
Razavi et al. (1990)	All items	---------	---------	
Caci et al. (2003)**	1,3,5,9,13	2,4,6,8,10,12	7,11,14	

*The three-factors are correlated in this model. ** Two models based on Caci et al. are tested, the second model removing item 10. ^+^Based on CFA of three independent samples of N = 894, 829 and 824, the total cohort in this study is 2,547. ^#^PCA: Principal Components Analysis; CFA: Confirmatory Factor Analysis. **FLI: Factor Loading Items. The HADS items loading on each model tested.

### Procedure

The first author administered the Chinese version of HADS to patients for self-completion. Demographic data and medical history were also obtained from patients and medical charts.

### Participants

One hundred and sixty patients with CHD were initially enrolled into the study of which 154 completed the questionnaires. The patients ranged in age from 38 to 86 years with a mean age of 60 years (SD = 10.37). One hundred and twenty (77.9%) patients were males. In terms of the clinical data, over two thirds of subjects were angina patients. The study was conducted in the general cardiovascular wards of two large university-based teaching hospitals in Xi'an City of China. Inclusion criteria were diagnoses of angina pectoris or myocardial infarction, no known psychiatric problems, and could understand Chinese. The study was conducted over a four-month period. The clinical ethical committee of the two university affiliated hospitals in Xi'an approved the study. Informed consent was obtained from all patients prior to commencement of the study.

## Results

The mean HADS anxiety (HADS-A) sub-scale score was 6.16 (SD 3.86) and the mean HADS depression (HADS-D) sub-scale score was 6.43 (SD 4.12). Based on Snaith and Zigmond's [29] interpretation of HADS-A and HADS-D scores of 8 or over, approximately one-third of the patients screened positive for anxiety (32%) and/or depression (35%). The results of the CFA are summarised in Table 2. and reveal that the two-factor model of Moorey et al. [25] and the three-factor model of Dunbar et al. [17] offered the best fit to the data. The model fit indices, which revealed identical values for both models, was highly acceptable by conventional model-fit criteria [30-33].

**Table 2:** Factor structure of the HADS determined by testing the fit of models derived from factor analysis. All χ^2 ^analyses were statistically significant at p < 0.01 (χ^2 ^degrees of freedom in parentheses).

**Model**	**WLSMVχ**^**2**^	**CFI**	**TLI**	**RMSEA**
Zigmond and Snaith (1983)	65.61 (33)	0.95	**0.97**	0.08
Moorey *et al. *(1991)	57.79 (32)	**0.96**	**0.97**	**0.07**
Caci *et al. *(2003) model 1	71.04 (32)	0.94	0.96	0.09
Caci *et al. *(2003) model 2^#^	73.92 (29)	0.93	0.95	1.00
Dunbar *et al. *(2000)	60.19 (33)	**0.96**	**0.97**	**0.07**
Friedman *et al. *(2001)	62.99 (32)	0.95	**0.97**	0.08
Razavi *et al. *(1990)	137.54 (30)	0.84	0.89	0.15

Note: Bold indicates best model fit as a function of model fit index criterion. Abbreviations: Weighted least-square with mean and variance correction (WLSMV); Comparative fit index (CFI); Tucker-Lewis Index (TLI); Root mean squared error of approximation (RMSEA). ^# ^Three-factor model 2 excludes item 10.

## Discussion

The finding of high proportions of Chinese CHD patients screening positive with anxiety and depression using the HADS is consistent with previous studies conducted in China or in western countries [3,5,34,35]

The CFA findings are in many respects, rather surprising. It has been suggested that the bi-dimensional underlying factor structure of the HADS observed in many early psychometric evaluations of this instrument is an artifact of the factor extraction method (exploratory factor analysis) and that the instrument is indeed tri-dimensional, a perspective supportive by more recent studies using CFA [16]. In studies of the HADS in patients with CHD using CFA, a clear advantage of three-factor models over two-factor models is consistently observed [18-20]. In the current study the best performing three-factor and two-factor models were equivalent in terms of model fit indices and offered a good fit to the data. It is interesting however to reflect that the best-performing two-factor model of Moorey et al. [25], represents a modification of the original model proposed by the instrument developers [7], suggesting that even in the context of a two-factor model, the traditional HADS scoring system may not be optimal. The best-performing three-factor model of Dunbar et al. [17] is an important observation because it represents a good performing model based on a cogent theoretical model of anxiety and depression. The observation of a well-fitting theoretical model when applied to clinical data is deemed to be a good test of the model.

Given the observation that tri-dimensional models offered a similar fit to the data as bi-dimensional models, the issue of scoring the instrument as a tri-dimensional instrument is worthy of discussion. It has been previously suggested that the HADS could be scored as a three sub-scale instrument [17]. However, such tri-dimensional scoring approaches that have been proposed are complex and time-consuming for busy practitioners to use routinely since they require factor scores to be regressed to calculate sub-scale scores [17]. A key operational rubric of the HADS is that it is a quickly administered and easily scored measure, therefore implementation of a complicated scoring system would be highly undesirable. Moreover, the extensive use of the HADS in clinical research over the last 20 years has led to the dissemination of several hundred publications reporting the HADS sub-scale means for a broad range of clinical groups. Adopting a tri-dimensional scoring approach would essentially remove this valuable reference data for comparative purposes in new research. Finally, the principle finding of the current study of no clear advantage of tri-dimensional models over bi-dimensional models would suggest that consideration of tri-dimensional scoring approaches is at the very best, highly premature.

The finding of virtually identical fit characteristics of the best performing two and three-factor models also raises the issue of conclusively defining the underlying factor structure of the HADS. The HADS clearly cannot be both bi-dimensional and tri-dimensional within the same data set and further clarification of the structure of the instrument is desirable since this may provide additional evidence not only on the limitations of the HADS, but also the development, enhancement and possible future revision of this widely used measure. The current study was limited by sample size and this may be an important factor in clarifying the relative performance of the competing models tested. It is worthy of note that a number of studies that have utilised factor analysis with large sample sizes have found a clear advantage in model fit of tri-dimensional models over the traditional anxiety/depression bi-dimensional model of the HADS [17,18,26]. Large sample sizes are generally desirable in confirmatory factor analysis and the conservative sample size of the current study may have contributed to the absence of differences in model fit between the best-fit two and three-factor models. Further research is necessary to address this particular issue conclusively.

Previous findings of the factor structure of the Chinese version of the HADS in Cantonese-speaking Chinese CHD patients in Hong Kong indicates clear and consistent superiority of three-factor models in fits to data [20]. One possibility that may account for the ambiguous factorial conclusions in the current investigation concerns the issue of translation. Translating English language instruments to Chinese language versions can be problematic in terms of establishing cultural and semantic equivalence [36,37].

The original validation of the Chinese version of the HADS [8] identified potential issues of case detection accuracy with the instrument. It is conceivable that problems of case detection accuracy may be artifactual of the original translation process, which may also explain inconsistencies between the underlying factor structure of the instrument between Mandarin-speaking Chinese CHD patients in the current study and those reported in Cantonese-speaking Chinese CHD patients.

It should be acknowledged that the current study had a number of limitations, in particular, the modest sample size and the absence of a comparison to a 'gold standard' such as a structured clinical interview to assess for anxiety and depressive disorder. Further research addressing these limitations is recommended.

## Authors' contributions

WW participated in the design of the study and assisted in the drafting of the manuscript. VL participated in the design of the study and assisted in the drafting of the manuscript. CM participated in the design of the study, performed the statistical analysis and assisted in the drafting of the manuscript.
